# A three‐microRNA signature identifies two subtypes of glioblastoma patients with different clinical outcomes

**DOI:** 10.1002/1878-0261.12047

**Published:** 2017-07-13

**Authors:** Giovanna Marziali, Mariachiara Buccarelli, Alessandro Giuliani, Ramona Ilari, Sveva Grande, Alessandra Palma, Quintino G. D'Alessandris, Maurizio Martini, Mauro Biffoni, Roberto Pallini, Lucia Ricci‐Vitiani

**Affiliations:** ^1^ Department of Hematology, Oncology and Molecular Medicine Istituto Superiore di Sanità Rome Italy; ^2^ Department of Environment and Health Istituto Superiore di Sanità Rome Italy; ^3^ Department of Technology and Health Istituto Superiore di Sanità Rome Italy; ^4^ Istituto Nazionale di Fisica Nucleare INFN Rome Italy; ^5^ Institute of Neurosurgery Università Cattolica del Sacro Cuore Rome Italy; ^6^ Institute of Pathology Università Cattolica del Sacro Cuore Rome Italy

**Keywords:** glioblastoma, glioblastoma stem‐like cells, microRNAs, patient stratification

## Abstract

Glioblastoma multiforme (GBM) is the most common and malignant primary brain tumor in adults, characterized by aggressive growth, limited response to therapy, and inexorable recurrence. Because of the extremely unfavorable prognosis of GBM, it is important to develop more effective diagnostic and therapeutic strategies based on biologically and clinically relevant patient stratification systems. Analyzing a collection of patient‐derived GBM stem‐like cells (GSCs) by gene expression profiling, nuclear magnetic resonance spectroscopy, and signal transduction pathway activation, we identified two GSC clusters characterized by different clinical features. Due to the widely documented role played by microRNAs (miRNAs) in the tumorigenesis process, in this study we explored whether these two GBM patient subtypes could also be discriminated by different miRNA signatures. Global miRNA expression pattern was analyzed by oblique principal component analysis and principal component analysis. By a combined inferential strategy on PCA results, we identified a reduced set of three miRNAs – miR‐23a, miR‐27a, and miR‐9* (miR‐9‐3p) – able to discriminate the proneural‐ and mesenchymal‐like GSC phenotypes as well as mesenchymal and proneural subtypes of primary GBM included in The Cancer Genome Atlas (TCGA) data set. Kaplan–Meier analysis showed a significant correlation between the selected miRNAs and overall survival in 429 GBM specimens from TCGA‐identifying patients who had an unfavorable outcome. The survival prognostic capability of the three‐miRNA signatures could have important implications for the understanding of the biology of GBM subtypes and could be useful in patient stratification to facilitate interpretation of results from clinical trials.

## 1 Introduction

Glioblastoma multiforme (GBM) is the most frequent and malignant primary adult brain tumor. Standard GBM treatment includes maximal safe surgical resection followed by combined radiotherapy and chemotherapy with the DNA‐methylating agent temozolomide (TMZ) (Stupp *et al*., 2005). Despite continuous improvements in the treatment for GBM during the past decade, these tumors are still associated with a poor prognosis and rare long‐term survival (Wen and Kesari, 2008).

Incurable GBM is characterized by uncontrolled cellular proliferation, robust angiogenesis, intense resistance to apoptosis, diffuse infiltration, propensity for necrosis, and genomic instability. Moreover, it exhibits a high degree of intra‐ and intertumor heterogeneity (Dunn *et al*., 2012).

Genomic profiling, chromosomal number variations, and abnormalities in DNA methylation have been used to define four subtypes of GBM, namely the proneural (oligodendrocytic signature), neural (oligodendrocytic, astrocytic, and neural signature), mesenchymal (cultured astroglial signature), and classical (astrocytic signature) subtypes (Verhaak *et al*., 2010).

Increasing evidence has led to the identification of a subpopulation of cells displaying stem‐like properties reminiscent of normal stem cells, called tumor‐initiating cells or GBM stem‐like cells (GSCs), that are believed to play a fundamental role in tumor resistance to chemo‐ or radiotherapy as well as in tumor recurrence (Singh *et al*., 2004). GSCs can be isolated to generate cell lines characterized by self‐renewing, multipotency, and high tumorigenic ability and are reported to recapitulate the genotype, gene expression patterns, and *in vivo* biology of human GBM more closely than many commonly utilized glioma cell lines (Ernst *et al*., 2009; Lee *et al*., 2006). The availability of cell lines that represent a more reliable model for understanding the biology of primary human tumors may help to identify cues for targeted therapies (Piccirillo *et al*., 2015).

One of the hallmarks of cancer is the defect in the regulatory circuits that control normal cell proliferation and homeostasis (Hanahan and Weinberg, 2011). Through the ability to regulate a large number of genes, microRNAs (miRNAs), a class of short noncoding RNAs, have been shown to control diverse oncogenic signaling pathways including cell proliferation, cell cycle regulation, apoptosis, invasion, glioma stem cell behavior, and angiogenesis. Dysregulated miRNAs are considered to be essential players in carcinogenesis and thus potential therapeutic targets (Mizoguchi *et al*., 2013). Deregulation of miRNAs can affect carcinogenesis if their target mRNAs are encoded by oncogenes or tumor suppressor genes (Lages *et al*., 2012); overexpression, silencing, or switching off specific miRNAs has been described in carcinogenesis of GBM (Brower *et al*., 2014; Floyd and Purow, 2014; Henriksen *et al*., 2014). Silencing or down‐regulation may result from deletion of a chromosomal region, epigenetic silencing, or defects in their biogenesis, whereas increased expression of mature miRNA may occur as a consequence of transcriptional activation or amplification of the miRNA‐encoding gene.

In an attempt to find druggable signaling pathways, we previously analyzed a collection of 19 patient‐derived GSCs by gene expression profiling, nuclear magnetic resonance (NMR) spectroscopy, and phosphoproteomic analysis of the signal transduction pathway (Marziali *et al*., 2016). We identified two GSC clusters, resembling the GSf and GSr groups described by Schulte (Schulte *et al*., 2011), although with distinct molecular signatures. Based on gene expression, NMR spectroscopy, and phosphoproteomic data, we found that the GSf‐like and GSr‐like clusters are characterized by a ‘proneural’‐ and ‘mesenchymal’‐like signature, respectively, similar to those described by Verhaak *et al*. (2010). Significant overlaps with the other two GBM subtypes (i.e., neural and classic) were not observed in our GSC collection. Phosphoproteomic analysis showed that the GSf‐like signature is characterized by a significant increase in SRC, mitogen‐activated protein kinase (MAPK), and insulin‐like growth factor receptor (IGF1‐R/IR), whereas GSr‐like lines displayed increased levels of phosphorylated proteins associated with the mammalian target of rapamycin (mTOR) pathway and a strong activation of downstream targets of the epidermal growth factor receptor (EGFR) (Marziali *et al*., 2016).

Classifying patients with GBM included in The Cancer Genome Atlas (TCGA) based on combined expression patterns of the two RPPA endpoints discriminating GSf‐ and GSr‐like phenotypes (i.e., SRC and RPS6, respectively), we showed that TCGA GBM patients with GSr‐like features display a significantly shorter overall survival (Marziali *et al*., 2016).

To further dissect the molecular biology of GSCs, in the present study we analyzed miRNA expression profile by microarray analysis to identify miRNAs differentially expressed between GSf‐ and GSr‐like sample groups. A reduced set of three miRNAs, able to discriminate GSf‐ and GSr‐like GSC phenotypes as well as mesenchymal and proneural GBM patient subtypes with different clinical outcomes, was identified.

## 2 Materials and methods

### 2.1 Clinical material and tumor characterization

Glioblastoma samples were harvested from 35 of 109 consecutive patients who underwent craniotomy at the Institute of Neurosurgery, Catholic University of Rome. All patients provided written informed consent according to research proposals approved by the Institutional Ethical Committee. Clinical and pathological features are summarized in Table S1. Patients were 38–80 years of age at diagnosis (median, 58 years); 26 were men and nine were women. The expression of the proliferation markers Ki67, phosphatase and tensin (PTEN), vascular endothelial growth factor, and EGFRvIII was characterized on tumor specimens by immunohistochemistry on deparaffinized sections as previously described (Martini *et al*., 2008, 2013; Montano *et al*., 2011; Pallini *et al*., 2008). *O* ^6^‐methylguanine‐DNA methyltransferase (MGMT) promoter methylation patterns by methylation‐specific PCR and isocitrate dehydrogenase 1/2 mutation state were assessed on genomic DNA extracted from paraffin‐embedded tissue as previously described (Horbinski *et al*., 2009; Pallini *et al*., 2008). Overall survival (OS) was calculated from the date of surgery where a diagnosis of GBM was established, to death. Progression‐free survival (PFS) was determined from the date of surgery until progression or death (Wen *et al*., 2010). After surgery, the patients received radiotherapy and concomitant TMZ followed by six cycles of adjuvant TMZ according to the Stupp protocol (Stupp *et al*., 2005; Wen *et al*., 2010).

Cox analysis was used for hazard ratio and 95% confidence interval determination. All *P*‐values are based on two‐tailed tests, and differences were considered significant when *P *<* *0.05. statview ver5.0 was used (SAS Institute, Cary, NC, USA).

### 2.2 Glioblastoma stem‐like cell cultures

Glioblastoma multiforme stem‐like cells were isolated through mechanical dissociation of the tumor tissue and cultured in a serum‐free medium supplemented with epidermal growth factor and basic fibroblast growth factor as previously described (Pallini *et al*., 2008). Cell lines actively proliferating required 3–4 weeks to be established. In these conditions, cells grow as clusters of undifferentiated cells, as indicated by morphology and expression of stem cell markers such as CD133 and SOX2 (Table S2). Stem cell marker expression was assessed by flow cytometry using a Canto analyzer (Becton Dickinson, Milan, Italy) using AC133‐PE antibody (Miltenyi Biotec, Bologna, Italy) and PerCP‐Cy™ 5.5 mouse anti‐Sox2 (BD, Becton Dickinson) for CD133 and Sox2, respectively. Viable cells were identified using 7‐amino actinomycin D (7AAD; Sigma Aldrich, St. Louis, MO, USA). To assess clonogenicity, viable cells were dispensed at different densities (1‐3‐10 cells per well) in 96‐well plates by cell sorting (FACS Aria; Becton Dickinson) (Table S2). After 10–14 days, wells with growing clones were enumerated, and results were analyzed by extreme limiting dilution assay (elda) software (Hu and Smyth, 2009). The *in vivo* tumorigenic potential of GBM neurospheres was assayed by intracranial or subcutaneous cell injection in immunocompromised mice in 30 of 37 GSC lines. GBM neurospheres were able to generate a tumor identical to the human tumor in antigen expression and histological tissue organization. GSC lines were validated by short tandem repeat DNA fingerprinting as previously described (Lulli *et al*., 2015).

### 2.3 Microarrays and real‐time PCR

To analyze GSC miRNA expression, total RNA was prepared using TRIzol Reagent (Invitrogen Life Technologies, Carlsbad, CA, USA). RNA was labeled and hybridized to the Agilent‐019118 array following the manufacturer's instructions. Microarray analysis was performed as previously described (Felli *et al*., 2010).

For real‐time PCR, 50 ng of RNA was reverse‐transcribed with TaqMan MicroRNA Reverse Transcription Kit (Applied Biosystems, Carlsbad, CA, USA). Real‐time PCR for miR‐23a‐3p (miRBase ID MIMAT0000078), miR‐27a‐3p (miRBase ID MIMAT0000084), and miR‐9‐3p (miRBase ID MIMAT0000442) was performed using TaqMan^®^ MicroRNA Assays protocol (assay ID 000399, ID 000408, ID 002231; Applied Biosystems). All reactions were run in duplicate. Normalization was performed by using RNU6B primer kit (ID 001093; Applied Biosystems). RT‐PCR was performed using an ABI Prism 7900 Sequence Detector (Applied Biosystems).

### 2.4 Statistical methods

In order to single out an effective miRNA signature for the GSf/GSr discrimination, we applied a four‐step strategy:

(i) clustering of the variables (miRNA expression profiles) by means of oblique principal component analysis (OPC) to single out (if any) a profile partition consistent with the GSf/GSr classification. OPC (Sethi, 1971) is a divisive clustering that partitions the variables of a data set into maximally internally correlated clusters. The partition stops when the ratio of intracluster to intercluster correlation reaches a maximum. If this purely data‐driven partition matches the GSf/GSr *a priori* classification, we have a proof‐of‐concept of the discrimination of the two subtypes in the miRNA space; (ii) application of principal component analysis (PCA) on the data set having miRNA as statistical units and samples (miRNA profiles) as variables. The seven miRNAs endowed with the highest scores on the *a posteriori* emerging discriminant component were selected. This data‐driven strategy allows for the elimination of overfitting problems (Napoletani *et al*., 2010) by concentrating on a purely unsupervised selection of miRNAs. After this step, we further refined our choice by the computation of (iii) mutual Pearson's correlations between selected miRNAs on a transposed (statistical units = cell lines; variables = selected miRNA species) subset of the original data matrix to eliminate redundant miRNA species (only one of strongly correlated pairs selected on the basis of their statistical significance as for GSf/GSr classification).

The last step (iv) was the application of linear discriminant analysis based on the three miRNA species previously selected on independent data sets in order to check their classification ability at both statistical (training set) and predictive (test set) levels.

## 3 Results

### 3.1 Multidimensional analysis of GSf‐ and GSr‐like miRNA profiles

Analyzing a collection of 19 GSC lines derived from 17 patients with GBM (from two of these, GSC lines from different tumor regions were isolated), by complementary molecular approaches, we recently identified two GSC clusters: one characterized by a proneural‐like phenotype (GSf‐like) and the other showing a mesenchymal‐like phenotype (GSr‐like) (Marziali *et al*., 2016).

To further examine the molecular biology of our GSC lines, we analyzed mature miRNA expression using a microarray platform. Following background subtraction and quartile normalization, the global miRNA expression pattern was analyzed by OPC analysis, a divisive variable clustering technique (Sethi, 1971). This analysis gave rise to a two‐cluster optimal solution (Table 1) that exactly mirrors the *a priori* GSf‐/GSr‐like classification except for line GSC#83.2. It is worth noting that, being OPC a completely unsupervised procedure and the GSf‐/GSr‐like label added *a posteriori*, the classification does not suffer from overfitting problems. The misclassified line (shown in italics in Table 1) lies on the border between the two clusters having an R‐square with its own cluster (own cluster R‐square = 0.79) of the same order of magnitude of the R‐square with the other class (R‐square = 0.72). The two‐cluster partition explains the 84.6% of total variance (proportion of variance explained, bolded in the Table 1) while, considering all the samples as part of the same group (one‐cluster solution), it accounts for 82.5% of total information. Thus, all the samples, besides their GSf‐/GSr‐like character, are largely invariant, pointing to a shared ideal miRNA profile characteristic of the tissue.

**Table 1:** Cluster summary for the two subtypes

Cluster	Members	Cluster variation	Variation explained	Proportion explained	Second Eigenvalue
1	11	11	9.074497	0.8250	0.6826
2	8	8	6.76652	0.8458	0.5199

Cluster	Variable	R‐squared with		1‐R**2	Variable
		^§^Own Cluster	^#^Next Closest	Ratio	Label
Cluster 1	GSC#1	0.9158	0.6729	0.2573	GSF
	GSC#28	0.9454	0.5663	0.1260	GSF
	GSC#62	0.8492	0.6188	0.3955	GSF
	GSC#23P	0.9251	0.5786	0.1777	GSF
	GSC#148	0.4918	0.3335	0.7625	GSF
	***GSC#83.2***	***0.7949***	**0.7244**	**0.7439**	***GSR***
	GSC#70	0.7282	0.2607	0.3676	GSF
	GSC#67	0.8538	0.5345	0.3142	GSF
	GSC#68	0.9416	0.7630	0.2464	GSF
	GSC#76	0.7078	0.6393	0.8101	GSF
	GSC#23C	0.9209	0.6639	0.2354	GSF
Cluster 2	GSC#30PT	0.7142	0.3783	0.4596	GSR
	GSC#151	0.8917	0.6500	0.3094	GSR
	GSC#147	0.8619	0.6964	0.4550	GSR
	GSC#83	0.8890	0.7076	0.3795	GSR
	GSC#61	0.8113	0.4935	0.3725	GSR
	GSC#30P	0.9034	0.5170	0.2000	GSR
	GSC#74	0.9037	0.5606	0.2191	GSR
	GSC#112	0.7912	0.7170	0.7379	GSR

Having verified the ability of the entire miRNA profile to correctly discriminate the two GBM subtypes, we checked for the existence of a single score able to select the best linear combination of miRNA species endowed with such discriminant ability. We faced this task by analyzing the same data set by a PCA, which allows for the projection of the initial 19 dimensions data set into a reduced space spanned by mutually independent axes (principal components) explaining the relevant (signal‐like) part of total variance of the system. Differently from OPC that partitions the variables into disjoint sets, PCA is a spectral method (Preisendorfer *et al*., 1988), and this implies that each miRNA species has a score relative to each component, while, at the same time, each sample has a peculiar correlation (loading) with all the extracted components.

**Figure 1:**
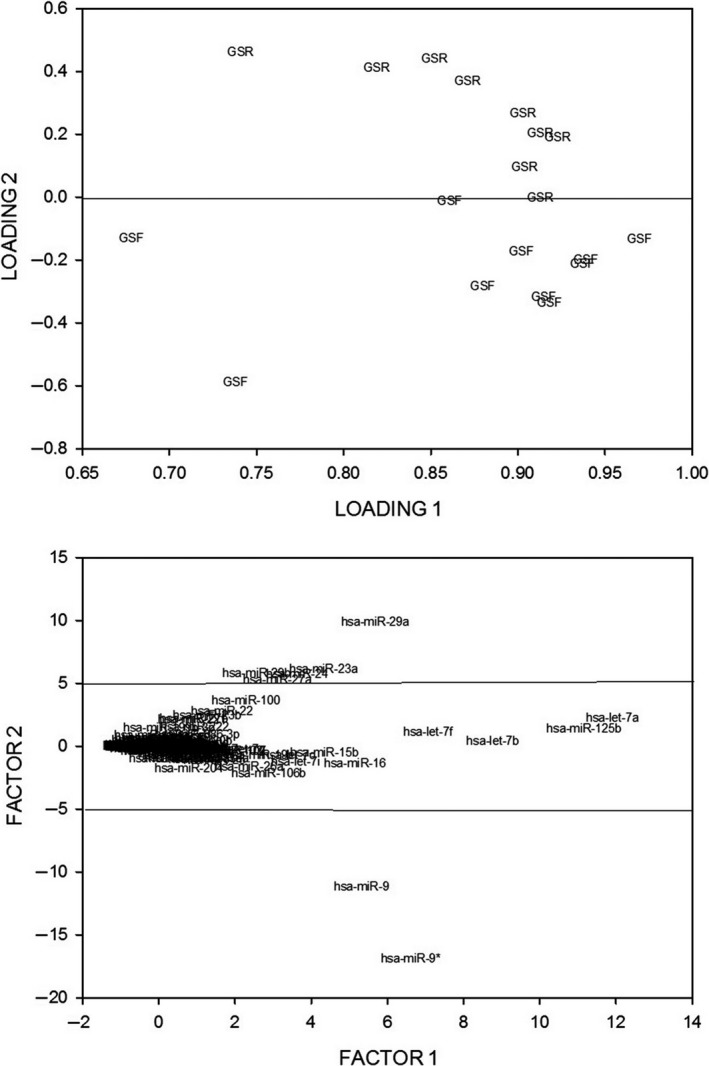
Principal component analysis of miRNA expression identifies two distinct clusters of GSC lines largely corresponding to the GSf‐like/GSr‐like classification described previously. Individual GSC samples (top) or miRNAs (bottom) are distributed into bivariate spaces spanned by the first two principal component loadings (top panel) and scores (bottom panel), respectively.

PCA showed a clear clustering of GSC lines and confirmed separation between GSf‐ and GSr‐like samples (Fig. 1, top panel). PCA suggests a two‐component solution as a *bona fide* reliable reconstruction of the 19‐sample space. The percent of variation explained by the first two components (Factor 1, Factor 2) is reported in Table 2A. The first component (Factor 1) explains the far major part of system variation (77% of total variance explained), while the second accounts for 8.5% of total variance. Globally, the two‐component solution accounts for 85% of total information. Pearson's correlation coefficients of the original variables (samples) with the extracted components constitute the factor‐loading pattern (Table 2B) and allow us to assign a meaning to the Factors.

**Table 2:** Percent of variation (A) and loading pattern (B) of the PCA two components

(A)	Eigenvalue	Difference	Proportion	Cumulative
1	14.5695598	12.9073361	0.7668	0.7668
2	1.6622236	0.9878260	0.0875	0.8543

(B)			Factor 1	Factor 2
GSC#1		GSF	0.93869	−0.19860
GSC#28		GSF	0.91768	−0.33740
GSC#62		GSF	0.90229	−0.17376
GSC#30PT		GSR	0.74159	0.46038
GSC#23P		GSF	0.91457	−0.31932
GSC#148		GSF	0.67795	−0.13114
GSC#83.2		GSR	0.91327	−0.00270
GSC#70		GSF	0.73815	−0.59035
GSC#67		GSF	0.87964	−0.28438
GSC#151		GSR	0.90307	0.26623
GSC#147		GSR	0.91298	0.20288
GSC#83		GSR	0.92311	0.19042
GSC#68		GSF	0.96986	−0.13348
GSC#76		GSF	0.86077	−0.01324
GSC#61		GSR	0.81959	0.41098
GSC#30P		GSR	0.85197	0.44001
GSC#74		GSR	0.87068	0.36793
GSC#23C		GSF	0.93719	−0.21187
GSC#112		GSR	0.90372	0.09592

Factor 1 is a ‘size’ component (Jolicoeur and Mosimann, 1960): all the samples have values close to unity loadings, in line with the existence of a ‘common miRNA ideal profile’ shared by all the samples, possibly due to the common tissue origin. Factor 2 is a ‘shape’ component mirroring the ‘GSf‐/GSr‐like’ axis: all the GSf‐like samples have negative loadings with Factor 2, while GSr‐like loadings are positive. Thus, miRNAs with high scores on Factor 2 point to the GSr‐like phenotype, whereas miRNAs with low scores identify the GSf‐like subtype. Results of PCA for both samples (loading space) and miRNAs (score space) are shown in Fig. 1.

### 3.2 A set of three miRNAs discriminates between GSf‐ and GSr‐like GSC subtypes

Focusing on miRNAs endowed with the maximal relevance of the discriminating component (Factor 2), we established a threshold (Fig. 1, bottom panel) corresponding to a score > 5 in absolute value to set a reduced signature. As principal components have by construction zero mean and unit standard deviation, they correspond to *z*‐scores; thus, a threshold of 5 corresponds to a *P* < 0.00001 as far as the influence of Factor 2 (i.e., the discriminating component) on the selected miRNA species is concerned. Seven miRNAs species emerged from the application of the above threshold. The Pearson correlation coefficients between these seven miRNA species were computed to eliminate redundant miRNAs (two miRNAs having near to unity correlation need not be both inserted in the signature, given they carry largely redundant information). Table 3 reports the miRNAs correlation matrix (bolded near to unity correlations). Correlations near to unity correspond to miRNAs already known to be structurally related (mature sequences of miR‐23a, miR‐27a, miR‐24‐2 derived from a common pri‐miRNA transcript encoded by miR‐23a/miR27a/miR‐24‐2 cluster on chromosome 19, miR‐9 and miR‐9*, originate from the opposite strand of the same precursor) (Liang *et al*., 2014). As expected, a statistically significant difference between the two groups was observed for all the seven miRNAs.

**Table 3:** Pearson's correlation coefficients

	Pearson's correlation coefficients, *N *= 19 *P *> |*r*| under H0: ρ = 0						
	hsa‐miR‐9*	hsa‐miR‐9	hsa‐miR‐27a	hsa‐miR‐24	hsa‐miR‐29b	hsa‐miR‐23a	hsa‐miR‐29a
hsa‐miR‐9*	1.00000	**0.99076**	−0.32515	−0.28202	−0.28583	−0.32931	−0.30315
hsa‐miR‐9*		< 0.0001	0.17440	0.24210	0.23550	0.16860	0.20710
hsa‐miR‐9	**0.99076**	1.00000	−0.35684	−0.29708	−0.32582	−0.35608	−0.33291
hsa‐miR‐9	< 0.0001		0.13370	0.21680	0.17340	0.13460	0.16370
hsa‐miR‐27a	−0.32515	−0.35684	1.00000	**0.92851**	0.41080	**0.98329**	0.40805
hsa‐miR‐27a	0.17440	0.13370		< 0.0001	0.08060	< 0.0001	0.08290
hsa‐miR‐24	−0.28202	−0.29708	**0.92851**	1.00000	0.32381	**0.96156**	0.31908
hsa‐miR‐24	0.24210	0.21680	< 0.0001		0.17620	< 0.0001	0.18300
hsa‐miR‐29b	−0.28583	−0.32582	0.41080	0.32381	1.00000	0.38931	**0.98087**
hsa‐miR‐29b	0.23550	0.17340	0.08060	0.17620		0.09940	< 0.0001
hsa‐miR‐23a	−0.32931	−0.35608	**0.98329**	**0.96156**	0.38931	1.00000	0.38926
hsa‐miR‐23a	0.16860	0.13460	< 0.0001	< 0.0001	0.09940		0.09950
hsa‐miR‐29a	−0.30315	−0.33291	0.40805	0.31908	**0.98087**	0.38926	1.00000
hsa‐miR‐29a	0.20710	0.16370	0.08290	0.18300	< 0.0001	0.09950	

In order to select, for each redundant pair, the ‘best representative’ species, miRNAs were ordered in terms of their *t*‐test statistics values (Table 4) and those with higher *t*‐value were chosen (bolded in Table 4). At the end of this procedure, a three‐miRNA signature (miR‐23a, miR‐27a, and miR‐9*) able to correctly cluster 18 of 19 GSC lines collection in two subtypes emerged (Table 5).

**Table 4:** Pearson's correlation coefficients ordered in terms of *t*‐test value

Variables	Method	Variances	DF	*t*‐Value	*P *>|*t*|
hsa‐miR‐9*	**Pooled**	**Equal**	**17**	**2.73**	**0.0143**
hsa‐miR‐9*	**Satterthwaite**	**Unequal**	**9.19**	**2.88**	**0.0178**
hsa‐miR‐9	Pooled	Equal	17	2.64	0.0173
hsa‐miR‐9	Satterthwaite	Unequal	9.69	2.78	0.0201
**hsa‐miR‐27a**	**Pooled**	**Equal**	**17**	−**3.17**	**0.0056**
**hsa‐miR‐27a**	**Satterthwaite**	**Unequal**	**8.74**	−**3.02**	**0.0151**
hsa‐miR‐24	Pooled	Equal	17	−2.66	0.0164
hsa‐miR‐24	Satterthwaite	Unequal	8.22	−2.52	0.0349
hsa‐miR‐29b	Pooled	Equal	17	−2.18	0.0439
hsa‐miR‐29b	Satterthwaite	Unequal	8.67	−2.07	0.0697
**hsa‐miR‐23a**	**Pooled**	**Equal**	**17**	−**3.43**	**0.0032**
**hsa‐miR‐23a**	**Satterthwaite**	**Unequal**	**8.97**	−**3.26**	**0.0098**
hsa‐miR‐29a	Pooled	Equal		−2.39	0.0287
hsa‐miR‐29a	Satterthwaite	Unequal	8.9	−2.27	0.0493

**Table 5:** Three‐miRNA signature clusterization of 19 GSC lines

	GSf‐like	GSr‐like	Total
GSf‐like	10	0	10
	100.00	0.00	100.00
GSr‐like	1	8	9
	11.11	88.89	100.00
Total	11	8	19
	57.89	42.11	100.00

To check the generalization ability of the selected signature, we analyzed the expression levels of miR‐23a, miR‐27a, and miR‐9* (now identified as miR‐9‐3p, www.mirbase.org) both in our sample set and in 18 newly established GSC lines by real‐time polymerase chain reaction (RT‐PCR) (Table S3), reaching a total of 37 GSC lines analyzed.

All the discriminant analyses were performed establishing a training set of 16 of 19 samples of known phenotype (seven GSr‐like and nine GSf‐like). This set was selected to build the linear discriminant function, which in turn was used for predicting an independent test set (*n* = 21; i.e., the 18 new GSC and the three remaining GSC lines previously analyzed). All the analyses made use of the same signature. Each discriminant analysis is reported in terms of its performance on both training and test sets by means of the relative confusion matrixes (Table 6). This analysis identified 7 of 21 GSC lines as GSr‐like subtype and the remaining as GSf‐like subtype.

**Table 6:** Three‐miRNA signature clusterization of 37 GSC lines

	GSr‐like	GSf‐like	Total
Training set			
GSr‐like	7	0	7
	100.00%	0.00%	
GSf‐like	0	9	9
	0.00%	100.00%	
Total	7	9	16
Test set			
GSr‐like	7	0	7
	100.00%	0.00%	
GSf‐like	1	13	14
	7.10%	92.90%	
Total	7	14	21

**Figure 2:**
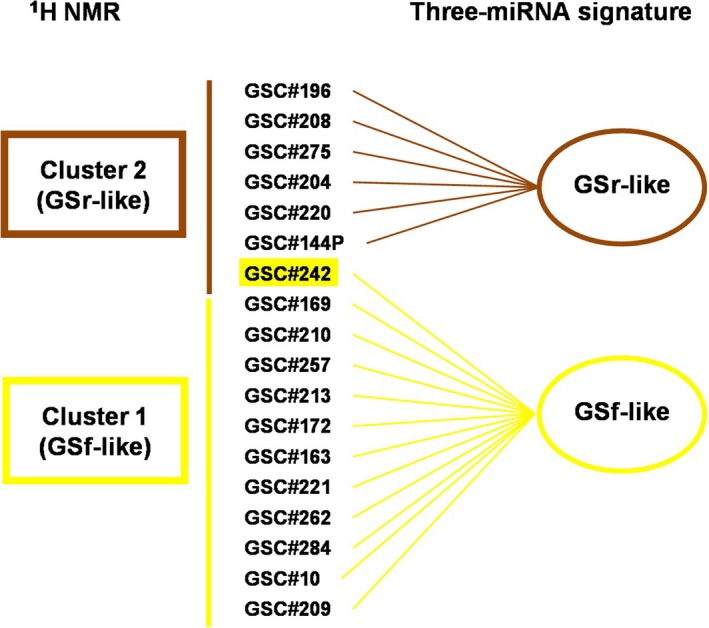
Classification into two clusters of GSC lines by miRNA signature reproduces the classification based on NMR analysis with the exception of one line.

To confirm the predictive potential of the three miRNAs to discriminate between GSf‐ and GSr‐like subtypes, the metabolite profiles of the 18 newly established GSC lines were analyzed by ^1^H NMR spectroscopy. We previously demonstrated that the GSf‐ and GSr‐like clusters are differentiated by NMR spectroscopy profiles: the GSf‐like, characterized by metabolites involved in the production of neurotransmitters such as NAA and GABA, with a prevalent neuronal metabolism; the GSr‐like, characterized by the lack of NAA and GABA and by high lipids, indicative of a prevalent astroglial‐like metabolism (Marziali *et al*., 2016). Unsupervised cluster analysis on all GSC lines clearly separated the samples in the two clusters (Fig. 2). According to NMR classification, the three‐miRNA signature was able to correctly clusterize 20 of 21 samples belonging to the test set confirming the reliability of the miRNA signature in classifying GSC subtypes (Table 6 and Fig. 2).

### 3.3 Patient clinical features and the three‐miRNA signature

Univariate analysis in all our patient cohort (*n *=* *35) showed that postoperative Karnofsky performance status (KPS) (< 70 *versus* ≥ 70), MGMT promoter methylation, and PTEN homolog expression were significantly associated with OS (*P *=* *0.0092, *P *=* *0.0167, and *P = *0.0284, respectively; log‐rank test; Table S4). Postoperative KPS ≥ 70, MGMT promoter methylation, and PTEN expression showed prognostic value also for PFS (*P *=* *0.0214, *P *=* *0.0440, and *P = *0.0485, respectively; log‐rank test; Table S4). To analyze possible association of miRNA signature with clinical and pathological parameters of the donor patients, we distinguished GBMs that generated GSf‐like cells (Group A, *n *=* *22) from those generating GSr‐like cells (Group B, *n *=* *13) (Table S5). Patients of group A and group B were homogeneous for the clinical and molecular features that hold prognostic value, such as age, extent of tumor resection, KPS, and MGMT methylation (Table S1). As previously described (Marziali *et al*., 2016), EGFRvIII expression was more frequent in tumors generating GSr‐like cultures than in those generating GSf‐like cultures. There were no significant differences in OS and PFS between GSf‐ and GSr‐like generating tumors (median OS = 9 and 8 months, median PFS = 5 and 4 months, *P *=* *0.5593 and 0.4921, respectively; log‐rank test; Table S5).

### 3.4 The three‐miRNA signature discriminates between proneural and mesenchymal subtypes in the TCGA cohort

Taking into account the potential bias of information obtained from our limited GSC samples, we tested the reliability of the signature in classifying GBM patients from completely independent sources such as the large cohort of patients with GBM from TCGA. Thus, we explored the expression of miR‐23a, miR‐27a, and miR‐9‐3p by using the Glioblastoma Bio Discovery Portal (gbm‐biodp software platform, http://gbm-biodp.nci.nih.gov). miRNA expression data were available for 429 patients, classified into four subtypes: proneural (P), neural (N), classical (C), and mesenchymal (M), as described by Verhaak *et al*. (2010). As previously reported (Marziali *et al*., 2016), the GSf‐like and GSr‐like clusters are characterized by a ‘proneural’‐ and a ‘mesenchymal‐like’ expression signature, respectively. Thus, we selected P (97 patients) and M (108 patients) from the 429 patients of the TCGA database and we performed the discriminant analyses according to the same scheme used for the 37 GSC lines described above. We defined a training set of 129 of 205 patients of known subtype (70 M and 59 P) to build the linear discriminant function, which in turn was used for predicting an independent test set (*n* = 76). As shown in Table 7, the analysis was able to correctly classify 30 of 38 M patients and 31 of 38 P patients, confirming the high predictive potential of the three‐miRNA signature.

**Table 7:** Three‐miRNA signature clusterization of patients with TCGA

	M	P	Total
Training set			
M	64	6	70
	91.4%	8.6%	
P	5	54	59
	8.5%	91.5%	
Total	69	60	129
Test set			
M	30	8	38
	78.9%	21.1%	
P	7	31	38
	18.4%	81.6%	
Total	37	39	76

**Figure 3:**
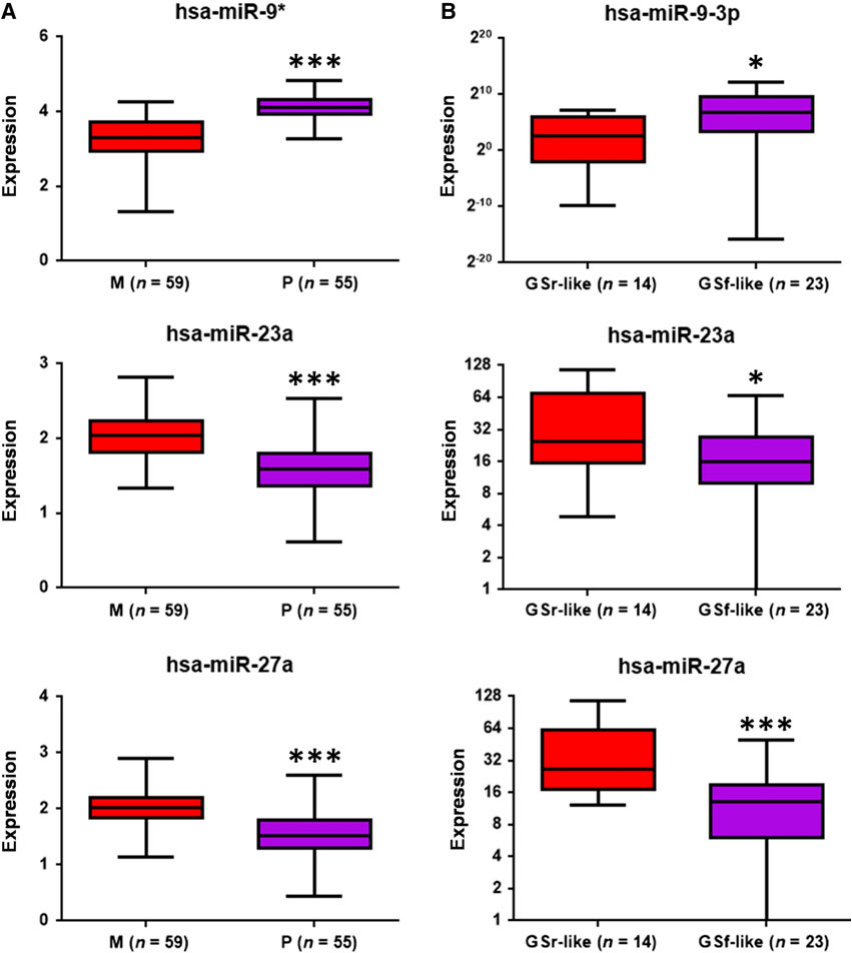
Box and whiskers plots of miR‐9‐3p (top), miR‐23a (center), and miR‐27a (bottom) expression in M and P subtype GBM samples extracted from TCGA (A) or in GSC lines (B). Numbers of samples in each group are indicated in brackets. The variability represents the range encompassing minimum and maximum values. * and *** indicate a significant (*P* < 0.05 and *P* < 0.001) difference between the two groups, respectively (unpaired *t*‐test, two‐tailed).

To further validate the ability of miR‐23a, miR‐27a, and miR‐9‐3p in discriminating between M and P GBM subtypes in the cohort of patients with TCGA, the gbm‐biodp software platform was used (Celiku *et al*., 2014). We found that both miR‐23a and 27a were significantly higher in the M *versus* P subtype, whereas miR‐9‐3p expression was significantly higher in the P *versus* M subtype, confirming a role for these three miRNAs in patient stratification (Fig. 3A). Similarly, using miR‐23a, miR‐27a, and miR‐9‐3p expression levels of our collection of GSC lines, we found that miR‐9‐3p expression was significantly higher in the GSf‐ *versus* GSr‐like subtype, whereas both miR‐23a and miR‐27a were significantly higher in GSr‐ *versus* GSf‐like subtype (Fig. 3B), confirming the ‘proneural’‐ and ‘mesenchymal‐like’ expression signature of the GSC subtype, respectively.

### 3.5 The three‐miRNA signature identifies two subtypes with different prognosis in the TCGA cohort of patients with GBM

In order to investigate the prognostic value of the three‐miRNA signature, we evaluated whether miRNA‐based classification correlated with clinical outcomes of the patients with TCGA.

**Figure 4:**
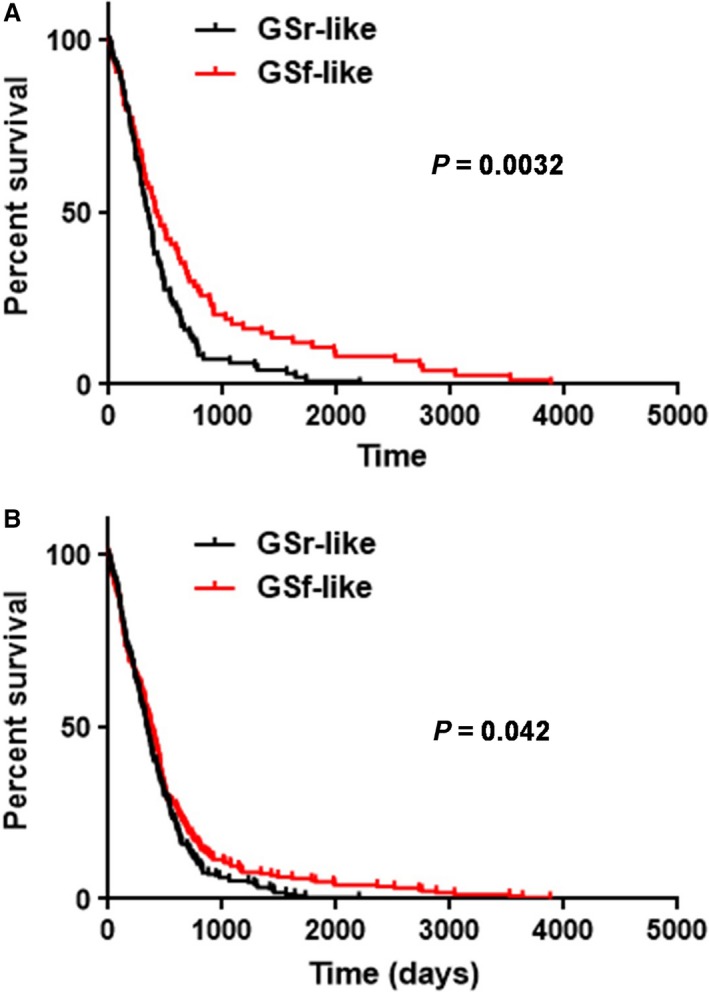
Kaplan–Meier analysis shows that among 169 patients with GBM from TCGA, prognosis was significantly worse in those classified as GSr‐like than in those classified as GSf‐like (*P* = 0.0032) (A). The classification based on miRNA expression applied to the whole cohort of 429 patients for whom survival and miRNA expression data were available, irrespective of the Verhaak subtype classification, revealed that the prognoses of the GSr‐like patients were significantly worse than the prognoses of those classified as GSf‐like (*P* = 0.042) (B). For this analysis, a training set of 177 of 429 patients of known subtype (93 M and 84 P) was defined to build the linear discriminant function for predicting the GSr‐ and GSf‐like subtypes of the independent test set of 252 patients.

We initially classified the patients categorized as M and P subtypes, according to Verhaak's criteria, for which survival data were available (*n* = 169 of 205). As shown in Fig. 4A, the prognoses of the patients classified as GSr‐like were significantly worse than those classified as GSf‐like (*P* = 0.0032). Then, to assess the generalized power of the three‐miRNA‐based classification for predicting patient clinical outcome, we analyzed the whole cohort of patients with TCGA, for which survival and miRNA expression data were available (*n* = 429), irrespective of the Verhaak subtype classification. A training set of 177 patients of known subtype (i.e., 93 M and 84 P) was defined to build the linear discriminant function used to classify the remaining 252 patients (independent test set). Patients with TCGA identified as C subtype were equally distributed between GSf‐ and GSr‐like subgroups, whereas the majority (2 : 1) of the N patients gathered in the GSf‐like subtype. This analysis classified 121 patients as GSf‐like and 131 as GSr‐like subtypes. Kaplan–Meier survival analysis (Fig. 4B) revealed that the prognoses of patients classified as GSr‐like were significantly worse than prognoses of those classified as GSf‐like (*P* = 0.042), indicating the three‐miRNA signature is significantly associated with survival.

### 3.6 Genes involved in cancer and neurodegenerative pathways are the preferential targets of the three‐miRNA signature

**Figure 5:**
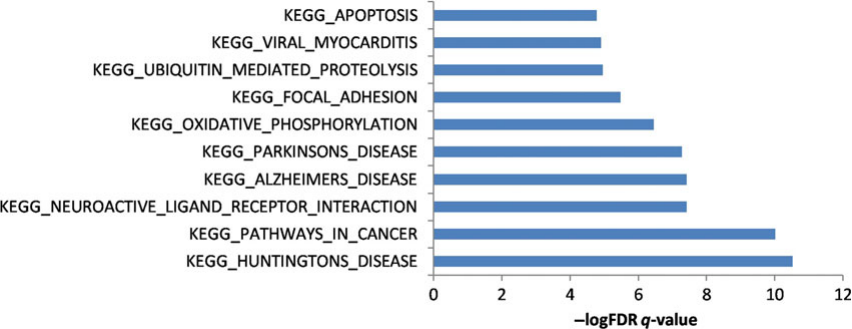
Pathway enrichment analysis of mRNA targets of the three miRNAs included in the signature indicates a significant association with cell survival, cancer, and cell adhesion but also with neurodegenerative diseases.

To explore the possible biological impact of miR‐23a, miR‐27a, and miR‐9‐3p in GBM, we derived the potential target genes from GB‐BioD. The lists of mRNAs correlating with selected miRNAs were then analyzed for pathway enrichment analysis by using the Kyoto Encyclopedia of Genes and Genomes (KEGG) present in MSigDB (GSEA online tool; http://www.broadinstitute.org/gsea/index.jsp). We found that the most significantly enriched pathways were related to cancer, apoptosis, or focal adhesion. Interestingly, a significant association with pathways involved in neurodegenerative disease emerged, suggesting that these three miRNAs may be associated with major neurodegenerative disorders such as Alzheimer's and Parkinson's diseases in addition to brain malignancies (Fig. 5 and Table S6).

## 4 Discussion

The identification of the key cellular pathways targeted by mutation during tumor progression is a high‐priority aim in cancer research. Using global gene expression profiling, several research groups have categorized GBM into distinct subtypes. The proneural (P) and mesenchymal (M) subtypes have been identified as representative classifiers and reported to have both prognostic and predictive values (Colman *et al*., 2010; Freije *et al*., 2004; Li *et al*., 2009; Phillips *et al*., 2006; Verhaak *et al*., 2010).

To date, classification of GBMs has not become part of clinical practice due mainly to the absence of a direct implication in the selection of a more appropriate therapy. However, identifying novel genetic signatures to gain better insights into the biology of cancers may help to develop novel therapies in the direction of precision medicine.

Several studies have reported miRNA profiles in GBM, highlighting the role of miRNAs in the progression of this disease and suggesting that miRNA signatures may be prognostic indicators of GBM and thus potentially, predict clinical outcome (Barbano *et al*., 2014; Fowler *et al*., 2011; Guan *et al*., 2015; Kim *et al*., 2011; Kouri *et al*., 2015; Lakomy *et al*., 2011; Pang *et al*., 2015; Zhang *et al*., 2016).

Here, we performed miRNA expression profiling of a collection of patient‐derived GSC culture and identified a three‐miRNA signature (miR‐23a‐3p, miR‐27a‐3p, and miR‐9‐3p) able to classify the cohort of TCGA GBM patients in two subgroups with significantly different overall survival.

In agreement with previous data, the expression of these three miRNAs is significantly altered in GBM compared with normal brain tissues (Rao *et al*., 2010; Visani *et al*., 2014).

The TCGA GBM data set showed decreased miR‐9‐3p and increased miR‐23a and miR‐27a expression preferentially in GBMs with a mesenchymal expression signature associated with a more invasive and aggressive behavior (Phillips *et al*., 2006). The same expression trends of these miRNAs were confirmed in our GSC samples.

Mature sequences of miR‐23a, miR‐27a, and miR‐24‐2 were derived from a common pri‐miRNA transcript encoded by mir‐23a/mir‐27a/mir‐24‐2 cluster on chromosome 19 (Liang *et al*., 2014).

Several studies have shown that miR‐23a is involved in the development and progression of multiple types of cancers, such as gastrointestinal, colorectal, esophageal squamous cell, and lung cancer (Chhabra *et al*., 2010). Recently, the up‐regulation of miR‐23a was found to be associated with glioma. It has been reported that miR‐23a acts as a key modulator in CREB/PTEN‐regulated gliomagenesis (Tan *et al*., 2012). Moreover, it promotes glioma cell proliferation via regulation of MXI1 (Xu *et al*., 2013), glioma cell invasion by inhibiting the expression of HOXD10 (Hu *et al*., 2013), and cell growth via targeting apoptotic protease activating factor‐1 (Lian *et al*., 2013).

The oncogenic role of miR‐27a has been confirmed by several experimental studies. miR‐27a was significantly up‐regulated in renal cell carcinoma (Nakata *et al*., 2015), in esophageal cancer (Wu *et al*., 2015), in gastric adenocarcinoma (Liu *et al*., 2009), and in breast cancer (Mertens‐Talcott *et al*., 2007). miR‐27a also contributes to oncogenesis by regulating cell cycle progression (Tian *et al*., 2014). It has recently been shown that miR‐27a is overexpressed both in human glioma samples and in cell lines (Yang *et al*., 2012).

miR‐9, found to be highly expressed in the brain of vertebrates, has been demonstrated to play a role in the development of the nervous system, regulating key genes in neurodevelopment by synergizing with its complementary miR‐9* (Schraivogel *et al*., 2011; Yoo *et al*., 2009). Both miRNAs may be important GBM mediators via regulation of multiple distinct signaling pathways and resistance to chemotherapy. Importantly, it has been reported that miR‐9 operate as a switch that regulates oligoneural *versus* mesenchymal decisions by suppressing mesenchymal differentiation through down‐regulation of JAK/STAT pathway (Kim *et al*., 2011). The same study also identified other miRNAs contributing to the phenotypic diversity of GBM subtypes, indicating that miRNAs may be useful for GBM classification allowing for the development of molecular treatment decisions and more accurate prognosis (Kim *et al*., 2011).

Recently, the EGFRvIII/Ras/PI3K/AKT axis has been shown to exert its tumorigenic influence through the specific inhibition of miR‐9 leading to the up‐regulation of the transcription factor FOXP1, thus providing a tumor growth advantage to EGFRvIII‐driven tumors (Gomez *et al*., 2014). Interestingly, among the molecular variables characterizing our GSC collection, EGFRvIII expression was significantly more frequent in tumors generating GSr‐like cultures than in those generating GSf‐like cultures. However, the median overall survival of the donor patients that generated GSr‐like cultures compared with that of the patients generating GSf‐like cultures was not significantly different. The lack of correlation with clinical outcome in our patients can be ascribed both to the small number of patients included in the study and to limiting factors related to GSC isolation: GSC cultures were obtained from only one‐third of the tumors. This implies that the GSC paradigm cannot be applied in a substantial fraction of patients suffering from GBM but mainly to more aggressive cases, because median overall and progression‐free survival were significantly shorter in tumors that generated GSC cultures compared with those that did not (Pallini *et al*., 2008).

Altogether, these data reinforce the concept that miRNAs are important actors in GBM carcinogenesis and thus potential therapeutic targets.

## 5 Conclusions

A three‐miRNA signature able to discriminate GSf‐ and GSr‐like GSC phenotypes as well as mesenchymal and proneural GBM patient subtypes was identified, confirming that miRNAs can be considered biomarkers for patient stratification. Even though clinical application of miRNAs either as potential therapeutics, as targets, or as biomarkers is still at the initial phase of development, several ongoing studies are investigating the predictive value of single or multiple miRNA levels in oncology. The three‐miRNA signature grouping patients with TCGA into two different classes with significant survival differences may be a promising prognostic tool that can improve the predictability of tumor aggressiveness. Thus, new knowledge about miRNAs in cancer has the potential to indicate new ways to stratify diseases and tailor an appropriate therapy to specific GBM patient subsets.

## Author contributions

LRV and GM conceived the study and wrote the manuscript; MBu performed experiments and analyzed data; RI performed experiments; AG performed statistical analysis; SG and AP performed NMR experiments; QGD'A provided samples; MM analyzed clinical data; MBi analyzed data and wrote the manuscript; RP provided and analyzed clinical data and wrote the manuscript. All authors read and approved the final manuscript.

## Supporting information


**Table S1.** Clinical parameters: comparison between groups.
**Table S2.** Stemness features of GSC lines (CD133 and Sox2 expression; estimated stem cell frequency evaluated by ELDA).
**Table S3.** Clusterization of GSC lines based on expression levels of miR‐23a, miR‐27a and miR‐9‐3p.
**Table S4.** Molecular parameters: comparison between groups.
**Table S5.** Univariate Kaplan–Meier analysis for prognosticators.
**Table S6.** Overlap of miRNA target genes with KEGG pathways.
