# Supplementary material for: A three‐microRNA signature identifies two subtypes of glioblastoma patients with different clinical outcomes
Source: Mol Oncol. 2017 Jul 13;11(9):1115–29. doi: 10.1002/1878-0261.12047 (PMC5579331; doi:10.1002/1878-0261.12047)
Supplement: Supplementary file 1 — Table S1. Clinical parameters: comparison between groups. Table S2. Stemness features of GSC lines (CD133 and Sox2 expression; estimated stem cell frequency evaluated by ELDA). Table S3. Clusterization of GSC lines based on expression levels of miR‐23a, miR‐27a and miR‐9‐3p. Table S4. Molecular parameters: comparison between groups. Table S5. Univariate Kaplan–Meier analysis for prognosticators. Table S6. Overlap of miRNA target genes with KEGG pathways. [file MOL2-11-1115-s001.pdf]

Supplementary Table 1. Clinical parameters: comparison between groups.

Parameter	Group A	Group B	*p*
Patients	22	13	NA
Age (mean ±SD)	58.0 ± 11.5	62.1 ± 10.1	0.2524^
Sex			
Males, *n*(%)	18/22 (81.8)	8/13 (61.5)	0.2427*
Females, *n*(%)	4/22 (18.2)	5/13 (38.5)	
Tumor Location			0.3602§
Frontal, *n*(%)	5/22 (22.7)	4/13 (30.7)	0.6978*
Temporal, *n*(%)	5/22 (22.7)	3/13 (23.1)	>0.9999*
Parietal, *n*(%)	10/22 (45.5)	3/13 (23.1)	0.2821*
Other, *n*(%)	2/22 (9.1)	3/13 (23.1)	0.3370*
Tumor diameter (mean ±SD) (cm)	4.6 ± 2.3	5.0 ± 1.2	0.6146^
Extent of resection			
Gross total removal, *n*(%)	16/22 (72.7)	10/13 (76.9)	>0.9999*
Partial removal, *n*(%)	6/22 (27.3)	3/13 (23.1)	
KPS, median (range)	70 (20-90)	70 (50-80)	0.8887^
MGMT Promoter			
Methylated	8/22 (36.4)	7/13 (53.8)	0.1952§
Un-methylated	14/22 (63.6)	6/13 (46.2)	
IDH1/2			
wt	16/17 (94.1)	9/9 (100)	NA
mt	1/17 (5.9)	0/9 (0)	

*, Fisher exact test; ^, Mann-Whithey U test; §, Chi-square test. NA, not applicable.

Supplementary Table 2. Stemness features of GSC lines (CD133 and Sox2 expression; estimated stem cell frequency evaluated by ELDA).

GSC #	CD133 (%)	SOX2 (%)	Estimated Stem Cell Frequency
1	95.8	95.2	4.3
23C	14.2	97.3	4.0
23P	14.3	89.2	4.0
62	82.7	92.9	9.3
68	1.2	90.9	32.2
67	0.2	80.0	5.6
70	17.3	38.0	17.6
76	1.8	88.4	7.2
148	94.8	94.3	14.5
169	6.6	47.4	5.2
210	95.9	90.4	75.8
257	6.2	75.0	13.5
213	56.3	92.0	10.5
172	2.7	94.8	17.9
163	1.2	89.2	2.3
221	64.3	91.1	11.1
262	10.1	81.2	7.3
284	79.1	91.5	4.2
10	23.0	94.9	5.8
28	1.4	82.0	3.8
120	83.8	84.0	31.9
209	0.3	91.4	5.4
30PT	0.0	47.4	6.3
30P	0.2	45.0	6.3
61	2.5	81.6	1.6
74	1.5	31.0	1.4
83	0.2	77.7	2.7
112	61.5	77.2	4.2
147	0.8	80.3	23.6
151	0.3	6.2	4.2
196	88.3	0.3	5.2
208	15.9	2.0	22.4
275	11.0	76.6	6.8
204	84.9	55.2	8.1
220	16.8	79.6	3.9
242	0.2	9.3	3.3
144P	13.2	45.7	7.5

Supplementary Table 3. Clusterization of GSC lines based on expression levels of miR-23a, miR-27a and miR-9-3p.

GSC #	hsa-miR-9-3p	hsa-miR-27a	hsa-miR-23a	Predefined Cluster	p (GSf-like)	p (GSr-like)	Extimated Cluster
1	222.7954262	0	0	GSf-like	0.991272642	0.008727358	GSf-like
23C	683.3850845	6.042614402	8.014418288	GSf-like	0.999677719	0.000322281	GSf-like
23P	109.4141108	13.04320851	15.83320486	GSf-like	0.85801189	0.14198811	GSf-like
62	67.9220925	5.58361421	14.45101447	GSf-like	0.999836776	0.000163224	GSf-like
68	64.02911339	6.114061732	14.31033404	GSf-like	0.999658929	0.000341071	GSf-like
67	1.324846762	2.433486528	4.594367606	GSf-like	0.979728222	0.020271778	GSf-like
70	179.9879408	7.195455175	13.48170111	GSf-like	0.99912987	0.00087013	GSf-like
76	9.970092751	1.604862104	1.547296091	GSf-like	0.920826203	0.079173797	GSf-like
148	19.00662212	1.964137632	1.827959985	GSf-like	0.912698687	0.087301313	GSf-like
169	10.6680914	16.30991701	31.41023107		0.999940383	5.96174E-05	GSf-like
210	22.24299039	7.810650235	10.08339133		0.915865365	0.084134635	GSf-like
257	15.1517594	18.95655752	24.34147643		0.765186071	0.234813929	GSf-like
213	2537.906927	21.76143432	27.3146424		0.999999999	1.03259E-09	GSf-like
172	4.63739E-05	13.30827775	32.71394065		0.999999201	7.99474E-07	GSf-like
163	561.0812037	22.22735143	26.65169733		0.976911463	0.023088537	GSf-like
221	3043.619518	10.28298623	16.68236807		1	2.16441E-13	GSf-like
262	1.79731E-05	21.67902026	63.74194527		1	1.83407E-13	GSf-like
284	4237.147735	17.54985938	12.35419655		1	1.56712E-12	GSf-like
10	4559.983706	14.16912163	24.57534341		1	1.60251E-19	GSf-like
28	3749.298701	32.70343253	34.13450018		1	5.63024E-11	GSf-like
120	808.4772341	9.222490043	11.40772377		0.999720391	0.000279609	GSf-like
209	128.005373	14.59738572	26.41930104		0.999819932	0.000180068	GSf-like
30PT	0.001078886	12.17471325	11.92880706	GSr-like	0.229394887	0.770605113	GSr-like
30P	0.522284982	25.12810303	27.5463935	GSr-like	0.035564867	0.964435133	GSr-like
61	123.8015206	43.80738253	47.80555391	GSr-like	0.000804619	0.999195381	GSr-like
74	5.387083378	61.90513871	68.65072089	GSr-like	7.6931E-06	0.999992307	GSr-like
83	62.11045456	66.84620277	80.20571031	GSr-like	0.000467285	0.999532715	GSr-like
112	148.5609935	27.88801372	16.65834562		9.61849E-07	0.999999038	GSr-like
147	9.542365852	17.70181501	18.46148433	GSr-like	0.10600566	0.89399434	GSr-like
151	6.713315708	115.3429331	116.3428616		2.31348E-15	1	GSr-like
196	0.004613743	13.9936	4.8227		0.000136825	0.999863175	GSr-like
208	0.058298753	22.16349898	21.2319		0.006673582	0.993326418	GSr-like
275	60.68376761	63.26064292	70.89165507		1.56143E-05	0.999984386	GSr-like
204	50.80846437	15.43251968	10.40997869		0.003475308	0.996524692	GSr-like
220	3.236229683	25.26222484	16.98746977		7.34844E-06	0.999992652	GSr-like
242	1.419823866	49.32700284	66.04589679		0.551852325	0.448147675	GSf-like
144P	0.300221285	27.17804853	30.38278597	GSr-like	0.034119422	0.965880578	GSr-like

Supplementary Table 4. Molecular parameters: comparison between groups.

Parameter	Group A	Group B	*p*
MGMT status			
methylated, *n* (%)	8/22 (36.4)	7/13 (53.8)	0.4810*
unmethylated, *n* (%)	14/22 (63.6)	6/13 (46.2)	
EGFRvIII status			
positive, *n* (%)	6/22 (27.3)	7/13 (53.8)	0.1568*
negative, *n* (%)	16/22 (72.7)	6/13 (46.2)	
PTEN status			
normal, *n* (%)	10/20 (50)	8/12 (66.7)	0.4709*
hypoexpressed, *n* (%)	10/20 (50)	4/12 (33.3)	
VEGF status			
normal, *n* (%)	5/22 (22.7)	1/13 (7.7)	0.3771*
hyperexpressed, *n* (%)	17/22 (77.3)	12/13 (92.3)	
Ki67 (mean ±SD)	29.3 ± 16.0	29.5 ± 11.4	0.8163^

*, Fisher exact test; ^, Mann-Whithey U test

Supplementary Table 5. Univariate Kaplan-Meier analysis for prognosticators.

Parameter	Median OS (mos)	*p*	Median PFS (mos)	*p*
Group A<br>Group B	9<br>8	0.5593	5<br>4	0.4921
Age > 60yrs<br>Age ≤ 60yrs	9<br>8	0.9441	4<br>5	0.5119
KPS > 70<br>KPS ≤ 70	12.5<br>5.5	0.0092	6<br>2.5	0.0214
Gross total removal<br>Partial removal	9<br>8	0.9903	5<br>4	0.6183
MGMT M<br>MGMT UM	14<br>8	0.0167	6<br>3	0.044
EGFRvIII positive<br>EGFRvIII negative	8<br>10.5	0.5389	4<br>5	0.4919
PTEN normal<br>PTEN hypoexpressed	8<br>9	0.0284	3<br>5	0.0485
VEGF normal<br>VEGF hyperexpressed	3<br>9	0.1459	1<br>5	0.1543
Ki67 > 20%<br>Ki67 ≤ 20%	9<br>8	0.0981	5<br>4	0.1915

Supplementary Table 6. Overlap of miRNA target genes with KEGG pathways.

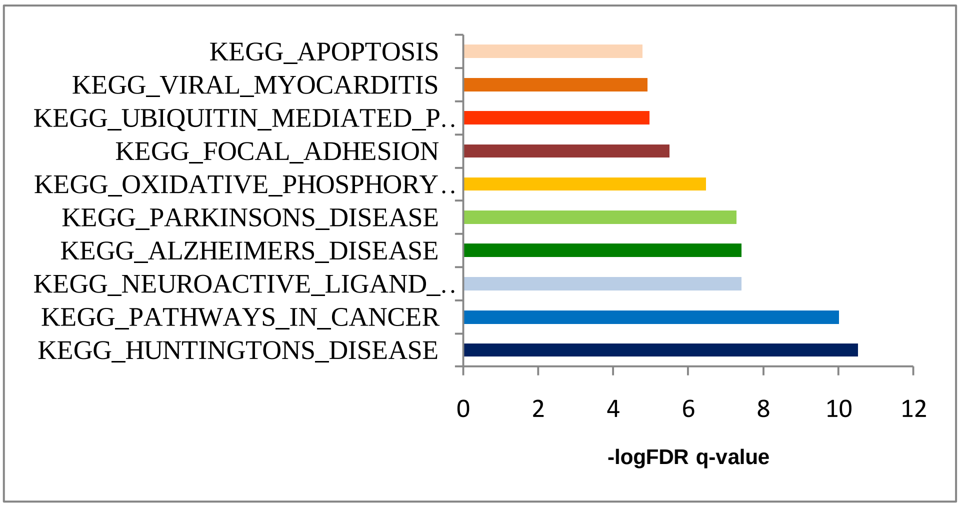

Entrez Gene Id	Gene Symbol
841	CASP8
581	BAX
5468	PPARG
3066	HDAC2
2904	GRIN2B
10975	UQCR11
1350	COX7C
1351	COX8A
7386	UQCRFS1
6392	SDHD
4695	NDUFA2
4696	NDUFA3
4701	NDUFA7
4705	NDUFA10
4717	NDUFC1
4719	NDUFS1
4724	NDUFS4
516	ATP5G1
518	ATP5G3
5331	PLCB3
3708	ITPR1
291	SLC25A4
292	SLC25A5
7416	VDAC1
83447	SLC25A31
8218	CLTCL1
5436	POLR2G
5437	POLR2H
6647	SOD1
6908	TBP
637	BID
8772	FADD
5290	PIK3CA
5291	PIK3CB
5154	PDGFA
7424	VEGFC
3082	HGF
3688	ITGB1
3675	ITGA3

2335	FN1										
10319	LAMC3										
3918	LAMC2										
3479	IGF1										
6502	SKP2										
356	FASLG										
4790	NFKB1										
7042	TGFB2										
5979	RET										
2248	FGF3										
2259	FGF14										
7185	TRAF1										
6258	RXRG										
1163	CKS1B										
7170	TPM3										
999	CDH1										
6934	TCF7L2										
1856	DVL2										
7175	TPR										
4286	MITF										
4292	MLH1										
4436	MSH2										
7184	HSP90B1										
112398	EGLN2										
5914	RARA										
5727	PTCH1										
154	ADRB2										
136	ADORA2B										
552	AVPR1A										
5025	P2RX4										
5724	PTAFR										
6915	TBXA2R										
2891	GRIA2										
2895	GRID2										
1081	CGA										
2554	GABRA1										
2555	GABRA2										
4142	MAS1										
7067	THRA										
10161	LPAR6										
1138	CHRNA5										
1142	CHRNB3										
187	APLNR										
2492	FSHR										
2560	GABRB1										
2912	GRM2										
2916	GRM6										
3061	HCRTR1										
3355	HTR1F										
4829	NMBR										
53637	S1PR5										
5540	PPYR1										
6344	SCTR										
64106	NPFFR1										
706	TSPO										
9248	GPR50										
6622	SNCA										
5664	PSEN2										

6263	RYR3										
25825	BACE2										
2861	GPR37										
7345	UCHL1										
51606	ATP6V1H										
529	ATP6V1E1										
534	ATP6V1G2										
10632	ATP5L										
5464	PPA1										
64077	LHPP										
7409	VAV1										
2318	FLNC										
3676	ITGA4										
3696	ITGB8										
6093	ROCK1										
5499	PPP1CA										
5500	PPP1CB										
4659	PPP1R12A										
5063	PAK3										
7060	THBS4										
1291	COL6A1										
1302	COL11A2										
11059	WWP1										
27339	PRPF19										
6477	SIAH1										
64682	ANAPC1										
8881	CDC16										
1643	DDB2										
8651	SOCS1										
9817	KEAP1										
22888	UBOX5										
389898	UBE2NL										
51366	UBR5										
55070	DET1										
63893	UBE2O										
7321	UBE2D1										
7323	UBE2D3										
8452	CUL3										
89910	UBE3B										
3106	HLA-B										
3135	HLA-G										
3122	HLA-DRA										
4628	MYH10										
3108	HLA-DMA										
1604	CD55										
1981	EIF4G1										
4620	MYH2										
4619	MYH1										
4622	MYH4										
4626	MYH8										
3562	IL3										
8795	TNFRSF10B										
4803	NGF										
8837	CFLAR										
843	CASP10										
51135	IRAK4										
